# Study of the mechanism by which dinaciclib induces apoptosis and cell cycle arrest of lymphoma Raji cells through a *CDK1*‐involved pathway

**DOI:** 10.1002/cam4.2324

**Published:** 2019-06-17

**Authors:** Huayan Zhao, Shenglei Li, Guannan Wang, Wugan Zhao, Dandan Zhang, Fang Wang, Wencai Li, Ling Sun

**Affiliations:** ^1^ Department of Hematology The First Affiliated Hospital of Zhengzhou University Zhengzhou China; ^2^ Department of Pathology The First Affiliated Hospital of Zhengzhou University Zhengzhou China

**Keywords:** *CDK1*, dinaciclib, lymphoma, Raji cell

## Abstract

**Objective:**

This study aimed to identify and evaluate the mechanism by which apoptosis and cell cycle arrest were induced by dinaciclib in lymphoma Raji cells.

**Methods:**

The colony formation assay was used to detect cell proliferation of Raji cells. Cell cycle arrest and cell apoptosis were determined by flow cytometry and TUNEL assays, respectively. Protein expression related to the Raji cell state was evaluated by Western blot. The Raji/Dinaciclib drug‐resistant cell line was established, where the regulating functions of *CDK1*‐involved pathway were verified. In addition, the effect of dinaciclib in vivo was examined in orthotopically implanted tumors in nude mice.

**Results:**

Cell apoptosis was induced, and DNA synthesis ability was decreased in a time‐dependent manner in dinaciclib‐treated lymphoma Raji cells. Furthermore, the cell cycle was found to be blocked in the G2/M Phase. Further study indicated that *CDK1*‐involved pathway played a key regulatory role in this process. It was revealed by cell transfection that the expression of cell cycle proteins was downregulated after treatment with dinaciclib through a *CDK1*‐involved pathway, which eventually led to apoptosis. Knockdown of *CDK1* restored the sensitivity of the Raji/Dinaciclib cells to dinaciclib. Xenograft model of nude mice showed that dinaciclib treatment in vivo could effectively inhibit tumor growth, consistent with the experiment results mentioned before.

**Conclusion:**

In this study, we clarified the mechanisms through which dinaciclib induces Raji cell apoptosis and blocks the cell cycle through a *CDK1*‐involved pathway, which supported that dinaciclib had potential values in the treatment of lymphoma.

## 1 INTRODUCTION

Lymphoma is one of the most common hematologic cancers in the USA.^1^ Different types of lymphomas differ from each other by both the types of malignant cells and the locations of tumor. Lymphomas most frequently originate from B cells and can be classified into non‐Hodgkin lymphomas (NHL) or Hodgkin lymphomas (HLs).^2^ Diffuse large B‐cell lymphomas accounts for half of the NHLs, followed by follicular lymphomas, marginal zone lymphomas, mediastinal lymphomas, and Burkitt's lymphomas (BLs).^3^ Earlier studies have described three clinical features of BL: endemic, sporadic, and immunodeficiency‐associated. Endemic BL referred to the cases occurring in Africa that are usually found in children with the involvement of multinodal and extranodal sites.^4^ Sporadic BL had no geographic predilection and accounts for 1%‐2% of all lymphomas in Western Europe and the USA.^5^ Immunodeficiency‐associated BL occurs mainly in patients infected with HIV, transplant recipients taking immunosuppressors, and individuals with congenital immunodeficiencies.^6^

Cyclin‐dependent kinases (CDKs) are serine/threonine kinases whose activity depends on a regulatory subunit, a cyclin.^7^ CDKs play important roles in the control of cell division and modulate transcription in response to several extra‐ and intracellular cues. The evolutionary expansion of the CDK family in mammals led to the division of CDKs into three cell cycle‐related subfamilies (Cdk1, Cdk4, and Cdk5) and five transcriptional subfamilies (Cdk7, Cdk8, Cdk9, Cdk11, and Cdk20).^7^ The CDK family is central to multiple signaling pathways controlling transcription and cell cycle progression.^7^ Cyclin‐dependent kinase 1 (CDK1) has recently been shown to promote replicative DNA synthesis and may contribute to chemoresistance. These findings provide novel insights into coordination between cell cycle regulation and DNA replication in the maintenance of genomic stability.^7^ Cdc2/cdk1 is a cyclin‐dependent protein kinase that controls the cell cycle entry from G2 to M phase.^7^

Dinaciclib (MK‐7965, SCH‐727965) is an orally administered small‐molecule CDK inhibitor (CDKI) that selectively inhibits important members of the CDK family (CDK1, CDK2, CDK5, and CDK9) at nanomolar concentrations.^8^ In preclinical studies, dinaciclib showed excellent anticancer efficacy, surpassing that of older CDKIs (eg, alvocidib (INN; also known as Flavopiridol or HMR‐1275) and seliciclib (roscovitine or CYC202)), and inhibited the growth of a broad spectrum of human cancer cell lines both in vitro and in xenograft models.^9^, ^10^ Dinaciclib is a CDK inhibitor with clinical potential in different cancers, including chronic lymphocytic leukemia,^11^ and dinaciclib treatment also increased accumulation of cells in G2/M phase and significantly induced apoptosis.^12^ Dinaciclib has been well‐tolerated in initial trials, and clinical efficacy has been observed in patients with chronic lymphocytic leukemia and solid tumors.^12^

Our study attempted to reveal the underlying mechanism by which dinaciclib induces apoptosis and cell cycle arrest in lymphoma Raji cells. Additionally, the *CDK1* pathway, which was involved in regulating the cell cycle and apoptosis in the lymphoma Raji cell line, was also investigated for its possible regulatory mechanism. In addition, we also present data showing how resistance can develop due to an upregulation of CDK1, and knockdown of CDK1 with siRNA restores sensitivity to dinaciclib. This research indicated that dinaciclib might act as an effective drug by downregulating CDK1 and bring new insight into the treatment of BL.

## 2 MATERIALS AND METHODS

### 2.1 Cell culture and dinaciclib‐resistance cell line establishment

Human lymphoma Raji cell lines were subscribed from BeNa Culture Collection, cultured under their explanatory memorandum, and maintained in RPMI‐1640 medium (Gibco, Grand Island, NY), with 10% fetal bovine serum (Gibco, Grand Island, NY) and 100 U/mL penicillin‐streptomycin supplemented to (Sigma‐Aldrich, St. Louis, MO).

A Raji/dinaciclib cell line was established by intermittent‐induced method of gradually increasing the concentration of dinaciclib (Selleck Chemicals, Houston, TX) into the Raji cell line in vitro with the dinaciclib concentration ranging from 4 to 20 μΜ. Then, a stable Raji cell line that was resistant to dinaciclib was obtained and harvested. All cell lines were incubated at 37°C, 5% CO_2_ in a moist environment.

### 2.2 Vector construction

Cells were seeded at a density of 1 × 10^6^ cells per well in 6‐well plates. PcDNA3.1‐*CDK1* and pcDNA3.1‐*CDK1* siRNA expression plasmids were constructed with the direction of pcDNA™3.1/V5‐His TOPO™ TA Expression Kit (Invitrogen, Carlsbad, CA). Then, cell transfection was conducted with Lipofectamine 3000 reagent (Invitrogen) according to the manufacturer's protocols (1 μg/2 × 10^5^ cells) in Opti‐MEM serum‐free medium. The transfection experiment was divided into two groups: the purified plasmid group and control group with empty vector plasmids. The sequences for the in vitro expansion of *CDK1* siRNA and *CDK1* cDNA were listed in Table 1.

**Table 1:** PCR primers

Compound	Forward primer 5’‐3’	Reverse primer 5’‐3’
CDK1 cDNA	GGAATTCCATGGAAGATTATACC	ATTAAGAAGATGTAGGGAATTCC
CDK1 siRNA	GGGTCAGCTCGCTACTCAAC	AAGTTTTTGACGTGGGATGC

### 2.3 Colony formation assay

The single‐cell suspensions of transfected cells were prepared (300 cells/mL), seeded into 6‐well plates with 20 μΜ dinaciclib, and cultured in growth media. When the cells produced a visible colony‐forming signal, cells were washed with PBS and stained with 0.1% crystal violet (Sigma‐Aldrich, St. Louis, MO) for 20 min. Finally, the crystal violet dye was washed with PBS. The number of colonies was observed and counted under the microscope (Olympus America Inc, Center Valley, PA). The percentage of colony formation was calculated by comparing cells that were cultivated in control group with those in the serum‐supplemented medium without dinaciclib.

### 2.4 Flow cytometry

Each group of Raji cells was diluted with 1 × AnnexinV binding Buffer (Bioco Laibo Technology Co., Ltd, Beijing, China) after transfection for 48 hours (8 × 10^5^cells/mL). Then, 20 μΜ dinaciclib (Selleck Chemicals), a FITC‐labeled enhanced bioluminescence‐based Annexin V probe (1:20), and 100 μg/mL of Phycoerythrin (PE) (1:100) were added in proper order to the 100 μL cell suspension. Upon incubation in the dark for 15 minutes at room temperature, 1 × Annexin V binding Buffer (400 μL) was added again. Next, the Raji cells were washed with PBS and fixed in 70% ethanol at 4°C overnight. Finally, cells were incubated with 20 μg/mL PI for 15 minutes. The DNA content was examined using flow cytometry (FCM), and the cell cycle analysis was performed using Cell Quest software (Becton Dickinson, USA).

### 2.5 TUNEL staining

A cell apoptosis assay was performed using 4', 6‐diamidino‐2‐phenylindole (DAPI) stain and Terminal‐deoxynucleotidyl Transferase Mediated Nick End Labeling (TUNEL) methods. The smears of Raji cells that were treated with 20μΜ dinaciclib (Selleck Chemicals, Houston, TX, USA) were fixed with 4% paraformaldehyde and incubated in 0.1% Triton X‐100 for 2 min. Next, 50 μL TUNEL reaction mixture was added to smears and were incubated with a lid for 1 hour at 37°C in darkness. Then, the Anti‐Fade Mounting Medium (Beyotime, Shanghai, China) with DAPI was added to the samples. Afterward, the samples were analyzed under a fluorescence microscope.

### 2.6 Western blot

Total proteins were isolated from the cells and tissues by RIPA lysis buffer (Beyotime, Shanghai, China). The BCA protein assay kit was used for measuring the protein concentration (Beyotime). The extracted total proteins were transferred to polyvinylidene difluoride membranes (Millipore, Billerica, MA) immediately following the separation operation by the SDS‐PAGE experiment. After the samples were blocked with tris‐buffered saline, Tween buffer containing 1% bovine serum albumin (Sigma‐Aldrich, St. Louis, MO) for 1 hour, the membrane was incubated with primary antibodies following the standard protocol. Then, the membrane was further incubated with the corresponding horseradish peroxidase (HRP)‐conjugated second antibody (1:2000; ZSbio, Beijing, China) for 2 hours at atmospheric temperature. The primary antibodies that were used in this study were the mouse polyclonal antibody to CDK1 (1:1000; Abcam, Cambridge, MA), rabbit polyclonal antibody to p‐CDK1 (1:500; Abcam), mouse polyclonal antibody to Cyclin D3 (1:1000; Abcam), rabbit polyclonal antibody to cleaved PRAP (1:1000; Abcam), mouse polyclonal antibody to cleaved caspase‐3 (1:1000; Abcam), and anti‐β‐actin (1:500, Abcam). The secondary antibodies included goat anti‐mouse IgG H&L (HRP) and goat anti‐rabbit IgG H&L (HRP) (1:2000, ZSbio, Beijing, China). The results of Western blot are grayscale analysis by ImageJ software. The expressions levels of proteins were normalized to β‐actin expression.

### 2.7 qRT‐PCR

Total RNA was isolated from cells or tissue samples by the TRIzol method (Invitrogen, Carlsbad, CA). Afterwards, according to the manufacturer's instructions, reverse transcription was conducted using the DNA Reverse Transcription Kit (Applied Biosystems, Foster City, CA) and the routine SYBR fluorescent staining was conducted for polymerase chain reaction (PCR) (Applied Biosystems, Foster City, CA). The glyceraldehyde‐phosphate‐3‐dehydrogenase activity (GAPDH) was used for an internal control to compare the expression of the detected mRNAs. The delta‐Ct (2^‐△△CT^) method was used to determine mRNA expression levels. The sequences of primers were listed in Table 2.

**Table 2:** Primers for qRT‐PCR

Gene	Forward primer 5’‐3’	Reverse primer 5’‐3’
CDK1	TCAGTCTTCAGGATGTGCTTAT	CTGTACCAGAGTGTTACTACCT
GAPDH	CGGAGTCAACGGATTTGGTCGTAT	AGCCTTCTCCATGGTGGTGAAGAC

### 2.8 Xenograft model in nude mice

A mouse xenograft model was used to evaluate the tumorigenesis and tumor progression in vivo. Four‐week‐old female BALB/c nude mice (weighted 18‐22 g) were obtained from Shanghai Experimental Animal Centre (Shanghai, China) and prepared for lymphoma Raji cell implantation under the conventional feeding. All experimental procedures involving animals got ethical approval of the protocols from the institutional committee of The First Affiliated Hospital of Zhengzhou University (following internationally established guidelines) before the research commenced. A subcutaneous injection of Raji cells (2 × 10^6^/mouse) was performed in the right axilla of the nude mice and dinaciclib (3 mg/kg; Selleck Chemicals) was intraperitoneally injected every other day. The tumor sizes were measured with a Vernier caliper every 5 days. In addition, the tumor volumes were computed according to the following formula: volume = length  × width^2^  × 0.5. Finally, mice were sacrificed 25 days after injection, and the tumor masses were harvested for subsequent examinations.

### 2.9 Hematoxylin and eosin staining

The tumor tissues of nude mice underwent fixation with stationary liquid and dehydration with gradient ethanol. Then, the tissue samples were transparentized by dimethyl benzene, embedded in paraffin, and cut into slices after solidification. Afterwards, the processed sections were dried off and then the histochemical stain with H&E (Hematoxylin solution and Eosin Y; Sigma‐Aldrich Shanghai Trading Co., Ltd., Shanghai, China) was performed in the following steps. It was stained with hematoxylin for 5 minutes, and then the tissue was washed with running water. The eosin stain was used for another 3 minutes. The dyed sections were observed by the microscope after which they were cleared in xylene and mounted with permanent mounting medium.

### 2.10 Immunohistochemistry staining

The expression pattern of CD33 was analyzed by immunohistochemistry (IHC). Paraffin‐embedded tissues were cut, dewaxed, and hydrated. After antigen retrieval by a microwave (citrate, pH 6) for 10 minutes, the endogenous peroxidase activity was blocked with 3% hydrogen peroxide (H_2_O_2_) in methanol for 10 minutes. The specimens were then incubated with normal nonimmune goat serum for 30 minutes. Rabbit polyclonal antibody to Ki67 (1:100; Abcam, Cambridge, MA) was used as the primary antibody with overnight incubation at 4°C. The sections were subsequently treated with goat anti‐rabbit IgG‐HRP (1:1000; Abcam, Cambridge, MA), followed by further incubation for 30 minutes at 37°C. The freshly prepared Diaminobenzidine (DAB) was used as a chromogenic agent, and the sections were lightly counterstained with hematoxylin for 15 seconds.

### 2.11 Statistics analysis

All of the assays described above in this research were run in triplicate, and the corresponding results were presented by their mean ± SD (standard deviation). GraphPad Prism 6 (La Jolla, CA) was utilized to perform the statistical analyses. The differences between groups (≤2) were analyzed using Student's *t* test, while a one‐way ANOVA was used for three or more (≥3) groups. The Pearson's correlation coefficient was analyzed to imply the dependence analysis between the expression levels of each gene. *P* < 0.05 was taken as an indication of statistical significance.

## 3 RESULTS

### 3.1 Dinaciclib treatment induced cell cycle arrest and increased apoptosis in lymphoma Raji cells

**Figure 1:**
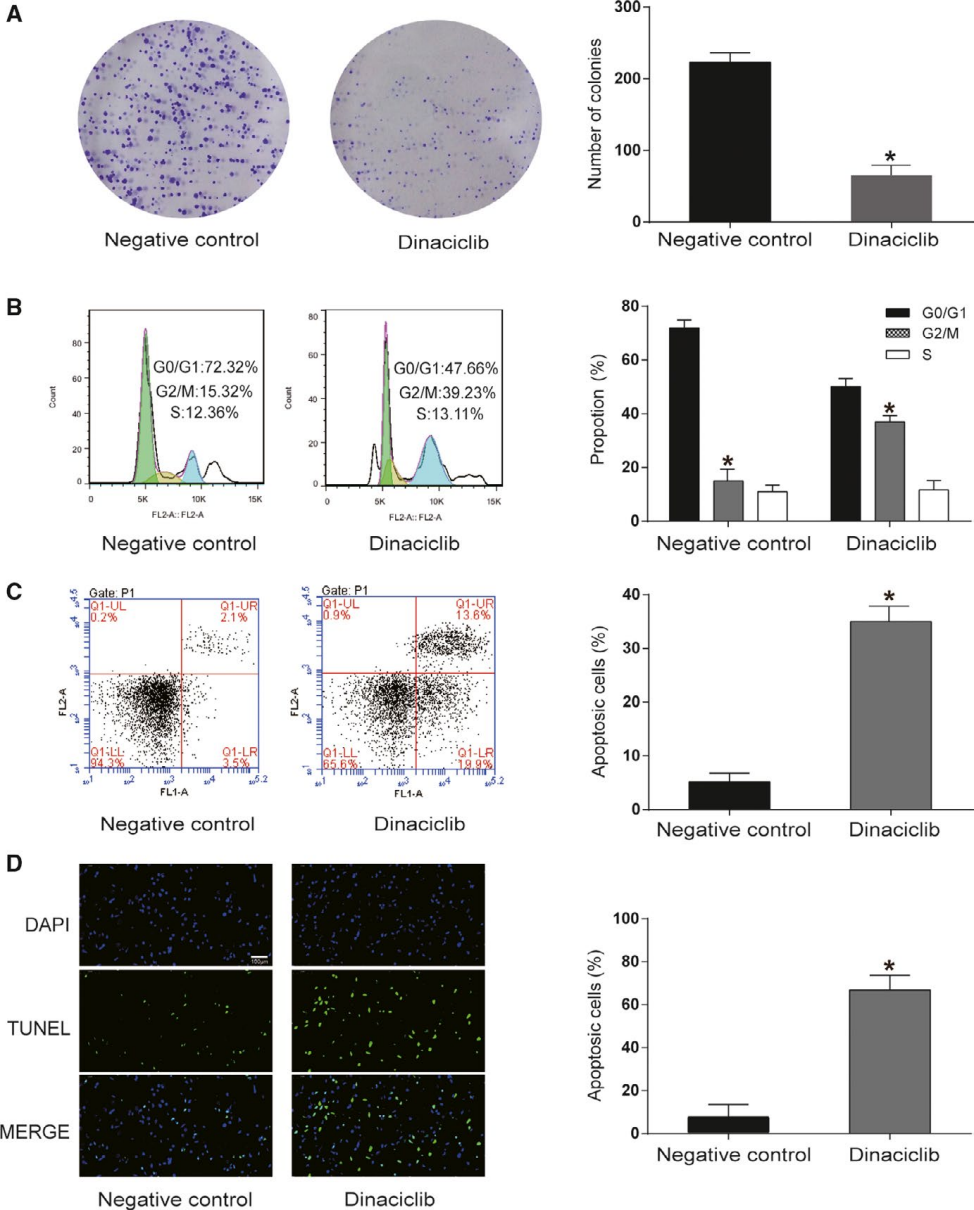
Dinaciclib induced cell cycle arrest and apoptosis in lymphoma cells. (A) Colony formation assay demonstrated that the multiplication capacity was significantly decreased in the Raji cell lines due to the dinaciclib treatment. (B) Cell cycle analysis performed by FCM indicated that dinaciclib could induce cell cycle arrest at the G2/M phase in Raji cell lines. (C) Cell apoptosis assay performed by FCM illustrated that dinaciclib could remarkably enhance the rate of apoptosis in the Raji cell lines. (D) TUNEL staining implied that the apoptosis rate was significantly increased in the Raji cell lines due to dinaciclib treatment. *P < 0.05, compared with the negative control group

To examine the biological functions of dinaciclib in Raji lymphoma cells, a series of in vitro experiments were conducted. The colony formation assay revealed that the proliferative ability of Raji cells was significantly suppressed following dinaciclib treatment (*P* < 0.05, Figure 1A). Cell cycle distributions were analyzed by FCM. The percentage of cells in the G2/M phase was increased in Raji cells that were treated with dinaciclib (*P* < 0.05, Figure 1B). To evaluate the mechanism of growth inhibition of Raji cell lines by dinaciclib, apoptosis of the Raji cells was detected by Annexin V‐FITC/PI double‐labeled flow cytometry. The apoptosis rate was the sum of the early apoptosis rate and late apoptosis rate. The result illuminated that dinaciclib could dramatically increase the apoptosis rate of Raji cells (*P* < 0.01, Figure 1C). In addition, the TUNEL assay supported the results we obtained from the cell apoptosis assay and demonstrated a higher apoptosis rate in Raji cells that were treated with dinaciclib (*P* < 0.05, Figure 1D). To verify these results, we repeated these experiments in BL cell lines, Ramos cells. The results were similar to those of the Raji cells. Dinaciclib inhibited Ramos cells proliferation, promoted cells cycle arrest in G2/M phase, and promoted cells apoptosis (Figure S1A‐D).

### 3.2 Dinaciclib regulated the signal pathways associated with the cell cycle and apoptosis in lymphoma Raji cells

**Figure 2:**
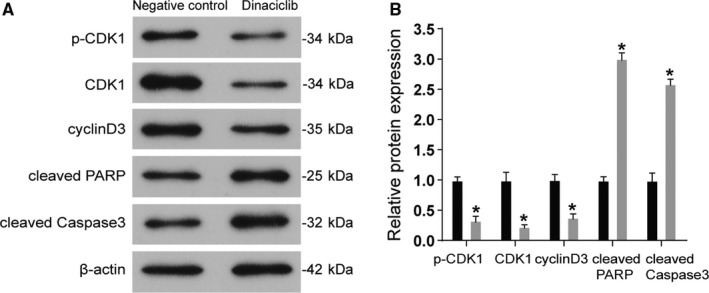
Dinaciclib regulated the signal pathway of cell cycle and apoptosis in lymphoma cells. (A) Western blot analysis illuminated that the expression of cell cycle‐related proteins p‐CDK1, CDK1, and Cyclin D3 was significantly decreased, whereas the expression of apoptosis‐related proteins, cleaved PRAP and cleaved Caspase‐3, were significantly increased in Raji cell lines treated with dinaciclib. (B) The statistic results of the Western blot analysis. *P < 0.05, compared with the negative control group

Western blotting was conducted to measure the expression levels of proteins related to cell cycle and apoptosis. The results revealed that the cell cycle‐related proteins, p‐CDK1, CDK1, and Cyclin D3, were significantly downregulated in Raji cells following dinaciclib treatment, indicating the effectiveness of dinaciclib as a CDK inhibitor. Simultaneously, the apoptosis‐related proteins, cleaved PRAP and cleaved Caspase‐3, were upregulated in Raji cells after dinaciclib treatment, and these results are consistent with the crucial role that is played by dinaciclib in activating signaling pathways that are involved in cell apoptosis (*P* < 0.05, Figure 2A,B). To verify these results, we repeated these experiments in Ramos cells. The result is consistent with the trend in Raji cells (Figure S1E).

### 3.3 *CDK1* regulated cell proliferation inhibition, cell cycle arrest, and dinaciclib‐induced cell apoptosis

**Figure 3:**
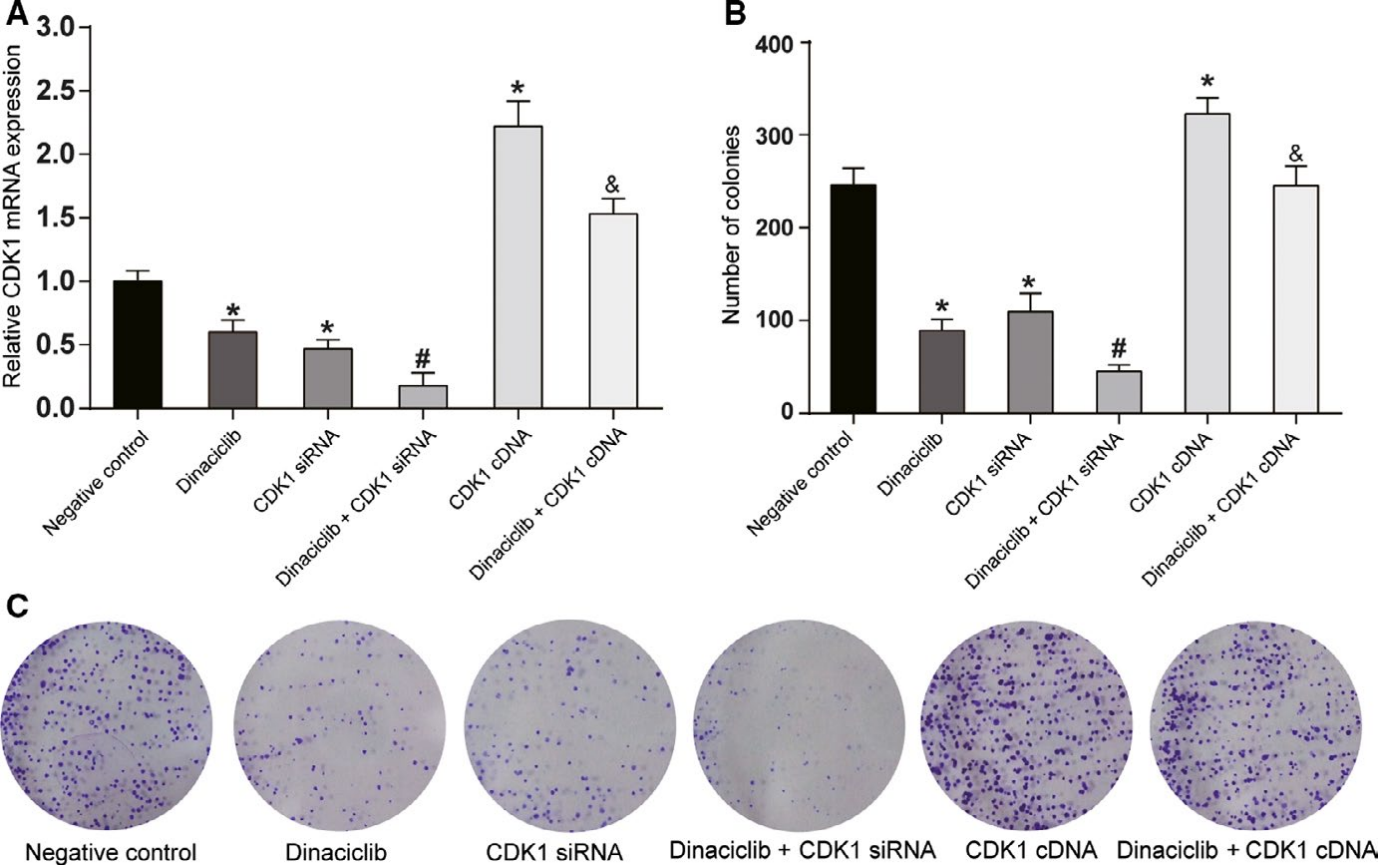
*CDK1* regulated the cell proliferation of Raji cell lines. (A) qRT‐PCR indicated that *CDK1* mRNA expression was significantly suppressed in Raji cell lines transfected with *CDK1* siRNA, whereas it was remarkably enhanced in Raji cell lines transfected with *CDK1* cDNA. (B‐C) The results of colony formation assay indicated that multiplication capacity was significantly inhibited in Raji cell lines that were transfected with *CDK1* siRNA. Furthermore, no remarkable difference was shown in Raji cell lines between the dinaciclib + *CDK1* cDNA group and the negative control group. *P < 0.05, compared with the negative control group; ^#^ *P* < 0.05, compared with the *CDK1* siRNA or Dinaciclib group; ^&^ *P* < 0.05, compared with the *CDK1* cDNA group

**Figure 4:**
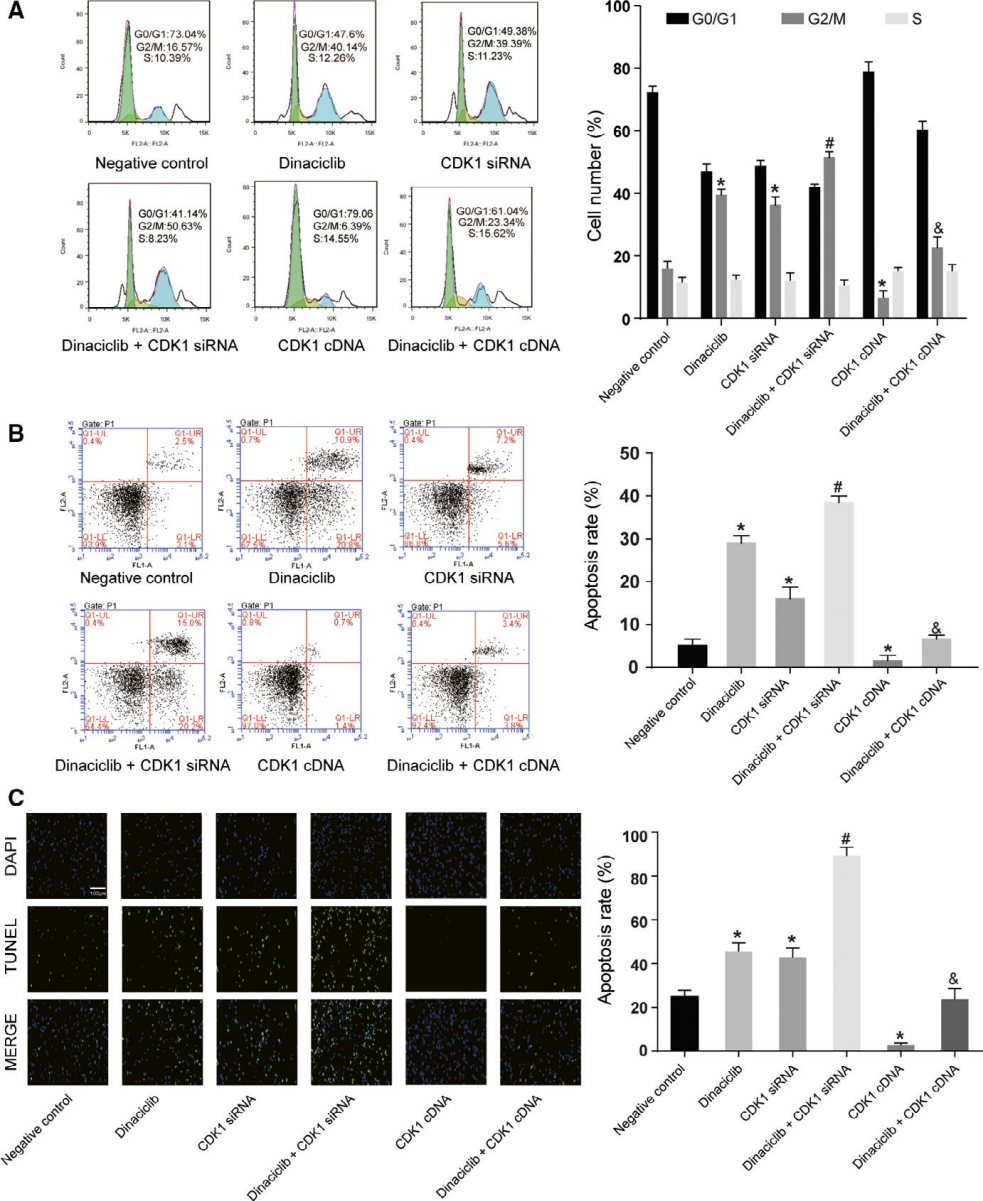
*CDK1* regulated the cell cycle and apoptosis in Raji cell lines. (A) Cell cycle assay indicated that dinaciclib and *CDK1* siRNA could induce cell cycle arrest at G2/M phase in Raji cell lines that were transfected with *CDK1* siRNA after dinaciclib treatment, whereas there was no notable difference showed in the Raji cell lines that were transfected with *CDK1* cDNA after dinaciclib treatment, compared with the negative control group. (B) Cell apoptosis assay illustrated that the apoptosis rate was significantly increased in Raji cell lines that were transfected with *CDK1* siRNA after dinaciclib treatment, whereas it was significantly decreased in the Raji cell lines that were transfected with *CDK1* cDNA after dinaciclib treatment. No remarkable difference was observed in Raji cell lines that were transfected with *CDK1* cDNA after dinaciclib treatment compared with those in the negative control group. (C) TUNEL staining method implied that these groups showed the same trend in apoptosis rates. *P < 0.05, compared with the negative control group; ^#^ *P* < 0.05, compared with the *CDK1* siRNA or Dinaciclib group; ^&^ *P* < 0.05, compared with the *CDK1* cDNA group

To investigate the effects of different expression levels of *CDK1* on the cell characteristics of Raji cells, either *CDK1* siRNA or *CDK1* cDNA was transfected into lymphoma Raji cells to downregulate or upregulate *CDK1* expression, respectively. qRT‐PCR and Western blotting revealed that transfection of *CDK1* siRNA and dinaciclib treatment could reduce CDK1 expression, and *CDK1* cDNA could increase CDK1 expression (*P* < 0.05, Figure 3A). The colony formation assay clarified that the proliferative ability of Raji cells was significantly suppressed with a low expression of *CDK1*, whereas the proliferative ability was strengthened with the overexpression of *CDK1* (*P* < 0.05, Figure 3B,C). The cell cycle assay indicated that Raji cells with low expression of *CDK1* were blocked in the G2/M phase, while those with a high expression of *CDK1* displayed reverse trend (*P* < 0.05, Figure 4A). The cell apoptosis assay and TUNEL staining showed that the apoptosis rate was dramatically increased in Raji cells with low *CDK1* expression, whereas a high *CDK1* expression had an inhibitory effect on lymphoma Raji cells (*P* < 0.05, Figure 4B,C). No remarkable difference in cell status was detected in Raji cells with an overexpression of *CDK1* after dinaciclib treatment compared with those in the negative control group. These results showed that Dinaciclib inhibited cell proliferation, promoted cell cycle arrest, and cell apoptosis through inhibiting CDK1. It indicated that overexpression of CDK1 could weak the effect of dinaciclib.

### 3.4 Knockdown of *CDK1* restored the sensitivity of the Raji/Dinaciclib cell line to Dinaciclib

**Figure 5:**
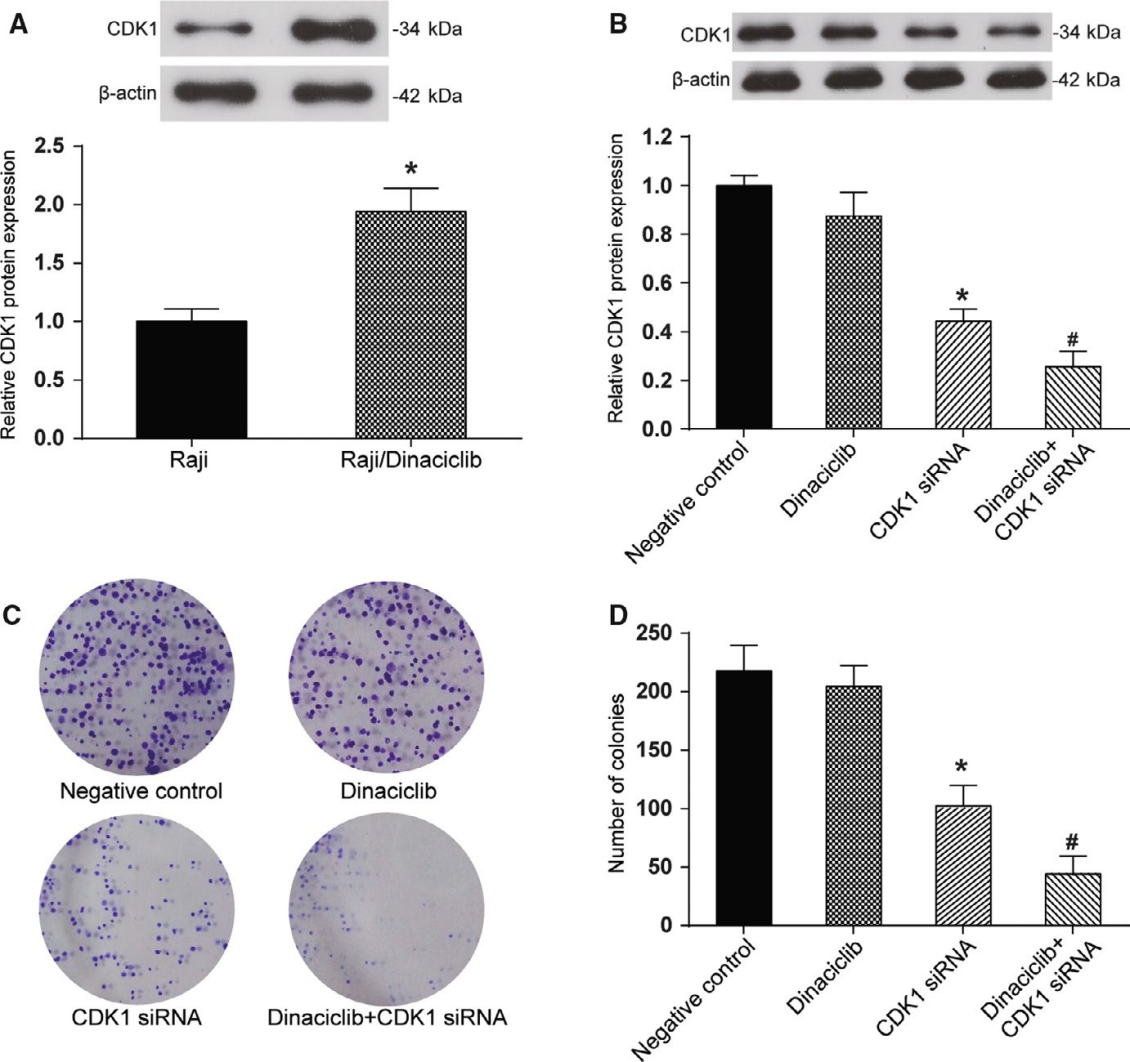
Knockdown of *CDK1* restored the sensitivity of drug‐resistant Raji cell lines to dinaciclib. (A) Western blot analysis indicated that CDK1 expression was significantly higher in the Raji/dinaciclib drug‐resistant cell lines. *P < 0.05, compared with the Raji group. (B) Western blot analysis indicated that the CDK1 expression was notably suppressed in the drug‐resistant cell lines that were transfected with *CDK1* siRNA after dinaciclib treatment. *P < 0.05, compared with the dinaciclib group; ^#^ *P* < 0.05, compared with the *CDK1* siRNA. (C, D) Colony formation assay indicated that the multiplication capacity was dramatically inhibited in the drug‐resistant cell lines that were transfected with *CDK1* siRNA after dinaciclib treatment. *P < 0.05, compared with the *CDK1* siRNA group; ^#^ *P* < 0.05, compared with the *CDK1* siRNA

**Figure 6:**
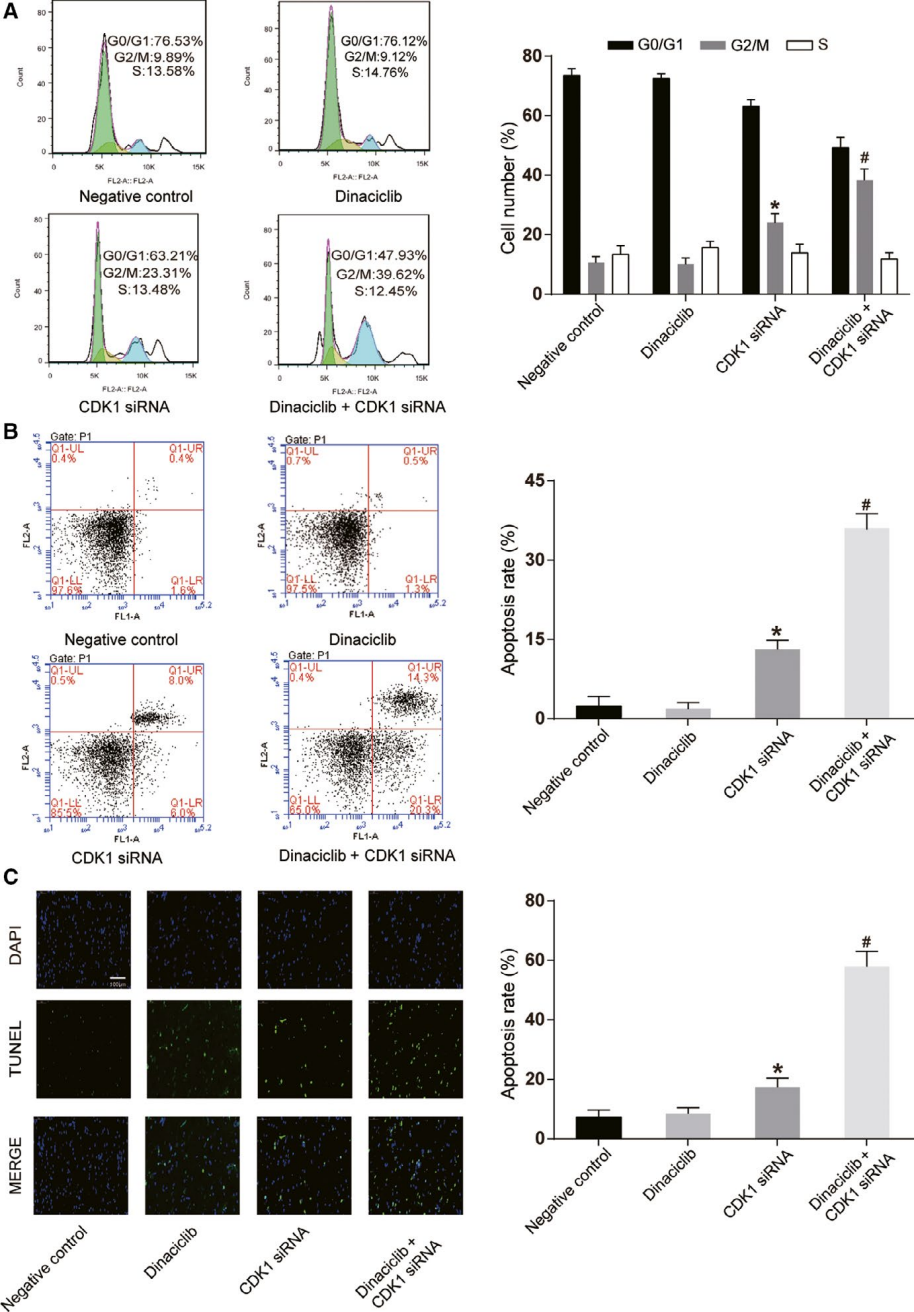
Knockdown of *CDK1* induced cell cycle arrest and apoptosis of drug‐resistant Raji cell lines. (A) Cell cycle assay implied that dinaciclib could induce cell cycle arrest at the G2/M phase in drug‐resistant Raji cell lines that were transfected with *CDK1* siRNA. (B) Cell apoptosis assay with FCM illustrated that dinaciclib could enhance apoptosis rates in drug‐resistant Raji cell lines after transfected with *CDK1* siRNA. (C)TUNEL staining demonstrated that dinaciclib could enhance apoptosis rates in drug‐resistant Raji cell lines after transfected with *CDK1* siRNA, confirming the former result. *P < 0.05, compared with the negative control group; ^#^ *P* < 0.05, compared with the *CDK1* siRNA groups

Western blotting was performed to measure the CDK1 expression levels in the drug‐resistant Raji/Dinaciclib cell line. The results indicated that CDK1 expression in the dinaciclib‐resistant cell lines was significantly higher than that in the primary Raji cell line (*P* < 0.05, Figure 5A). CDK1 siRNA was transfected to knock down the expression of CDK1 in the drug‐resistant cell line (*P* < 0.05), where dinaciclib treatment showed little influence on CDK1 expression (Figure 5B). The colony formation assay revealed that the proliferative ability of the Raji/dinaciclib cell line that was transfected with *CDK1* siRNA was significantly suppressed after dinaciclib treatment (*P* < 0.01), while dinaciclib had no remarkable impact on the dinaciclib‐resistant Raji cells without *CDK1* siRNA transfection (Figure 5C,D). The cell cycle assay indicated that the percentage of cells in G2/M phase was increased in Raji/Dinaciclib cell line that was transfected with *CDK1* siRNA after dinaciclib treatment (*P* < 0.01, Figure 6A). In addition, the cell apoptosis assay and TUNEL staining showed that the apoptosis rate was significantly increased in dinaciclib‐resistant Raji cells that were transfected with *CDK1* siRNA after dinaciclib treatment (*P* < 0.01, Figure 6B,C), indicating that knockdown of *CDK1* could reverse drug‐resistance to dinaciclib in Raji cells.

### 3.5 Dinaciclib suppressed tumor growth* in vivo*

**Figure 7:**
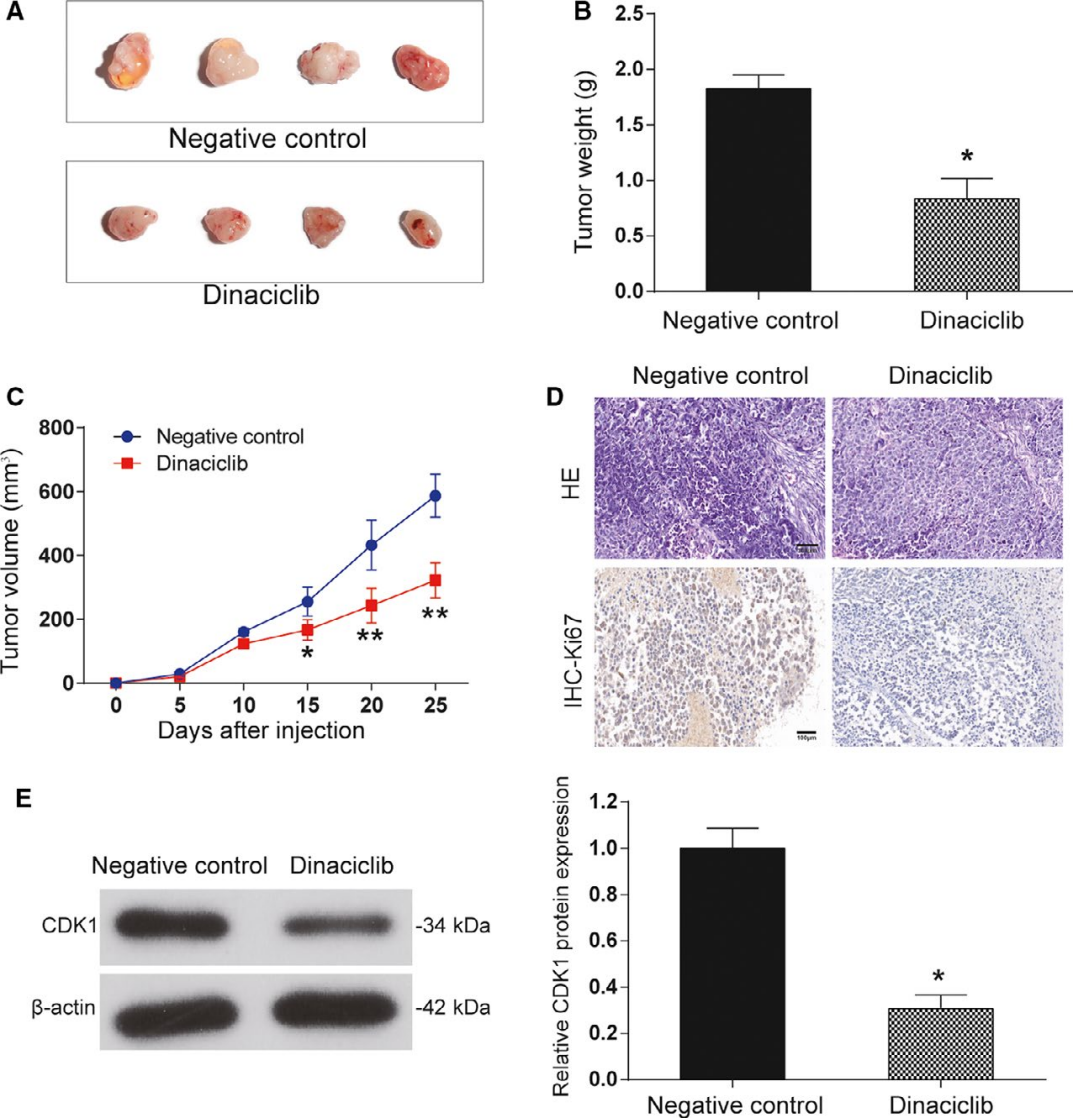
Dinaciclib suppressed tumor growth in vivo. (A) Representative images of tumors that formed in nude mice after they received subcutaneous injections of Raji cell lines that were or were not treated with dinaciclib. (B) Tumor weight chart of female nude mice with Raji cell lines implanted into the right axilla. (C) Tumor growth curve of female nude mice with Raji cell lines implanted into the right axilla. (D) HE staining indicated that the apoptosis rate of tumor tissues increased after dinaciclib treatment compared with the negative control group, whereas IHC staining implied the growth rate of tumor tissues decreased after dinaciclib treatment compared with the negative control group. (E) Western blot analysis revealed that CDK1 expression was significantly inhibited after dinaciclib treatment in vivo. *P < 0.05, **P < 0.01, compared with the negative control group

To further explore the effects of dinaciclib in lymphoma in vivo, xenograft models in nude mice were successfully established. When they were dissected at the end of the assay (on the 25th day), the tumors were markedly smaller and lighter in dinaciclib group compared with those in the negative control group (*P* < 0.05, Figure 7A‐C). The tumor tissues were subjected to H&E staining to detect cell apoptosis, and IHC analysis for the detection of the cell proliferation marker Ki67. The dinaciclib‐treated group showed that apoptotic cells were significantly increased in H&E staining and IHC staining of Ki67 illustrated a lower expression compared to the control group (Figure 7D). The findings indicated that dinaciclib could efficiently induce tumor apoptosis and thus regulate tumor progression. Accordingly, a Western blot analysis was performed on these extracted tumor tissues, and it demonstrated a remarkable decrease of both CDK1 expression in the dinaciclib group, confirming the preceding results (*P* < 0.05, Figure 7E). These results presented that dinaciclib might be a promising target for the treatment of malignant lymphoma through CDK1.

## 4 DISCUSSION

Four types of malignant lymphoma, including diffuse large B‐cell lymphoma, follicular‐lymphoma, Hodgkin's‐lymphoma, and mantle cell lymphoma, constituted more than half of all new lymphoma cases occurring worldwide.^13^ BL, a highly aggressive B‐cell NHL, is characterized by the activation of the most frequent amplified oncogene in human cancers (MYC) oncogene along with other genetic and epigenetic factors.^14^ Efforts have been made to improve the current methods of therapy for this rapidly growing type of neoplasm and for these therapies to have minimal treatment‐associated toxicities.^15^

CDKs are potential cancer therapeutic targets because of their critical role in promoting cell growth.^16^ Dysregulation of CDKs is regarded as hallmark events for nearly all types of human cancers, and they were critical regulators of cell cycle progression and RNA transcription.^17^ For instance, given the clinical significance of *CDK1* expression, it might serve as a biological marker for the degree of malignancy and the performance for oral squamous cell carcinomas.^18^ In addition, *CDK1* appeared to be related to chemoresistance in epithelial ovarian cancer.^19^ The development of techniques such as complete RNA sequencing has clarified some of the essential pathways that are activated in the pathogenesis of BL, among which *CDK1* has acquired wide attention. Phospho‐p70S6K and *CDC2/CDK1* have been investigated as potential therapeutic targets for diffuse large B‐cell lymphoma.^20^ Furthermore, a phase II, single‐arm, open‐label, multicenter study evaluated the efficacy and safety of P276‐00, another CDKI, in patients with relapsed or refractory mantle cell lymphoma.^21^ Together, these findings suggested that CDK1 could provide novel opportunities for improving the current therapeutic treatments of BL. Dinaciclib (SCH 727965) is a novel CDK inhibitor (CDK 1/2/5/9) currently under clinical evaluation for the treatment of advanced malignancies.^16^ Dinaciclib had been revealed in previous findings with significant influence on a variety of cancers in vitro and in vivo.^22^ For example, Chen *et al* found that Dinaciclib could synergize with cisplatin potently affecting the status of ovarian cancer in preclinical models.^23^ Dinaciclib showed encouraging single‐agent activity in myelomas,^12^ and its potential use in the treatment of breast cancer was clinically tested.^22^

In our study, we first found the molecular mechanism about the role of dinaciclib/*CDK1* in BL, which might offer potential for improved treatment and diagnostic strategies for this disease. We investigated the anticancer effects of dinaciclib on lymphoma Raji cells. Dinaciclib alone actively induced cell proliferation inhibition, cell cycle arrest, and increases in the apoptotic together with changes in expression of cell cycle‐ and cell apoptosis‐related proteins such as CDK1, CyclinD3, cleaved PARP, and cleaved Caspase‐3. Dinaciclib also inhibited tumor growth in xenograft nude mice that were subcutaneously injected BL Raji cells. The combination of dinaciclib treatment and *CDK1* cDNA transfection could counteract the dinaciclib‐induced effects on cell cycle arrest and apoptosis and elevated the resistance of the Raji/dinaciclib cell line to dinaciclib. Moreover, knockdown of *CDK1* restore the sensitivity of the Raji/dinaciclib cell lines to Dinaciclib, indicating that *CDK1* might be a promising target for treatment of BL.

A number of other studies have also investigated the mechanism of Dinaciclib in lymphoma. G P Gregory et al showed that Dinaciclib inhibits CDK9 and is highly effective against MYC‐driven B‐cell lymphoma by selectively inhibiting key MYC targets including McL‐1.^24^ Also, Colomer et al reported that Dinaciclib can treat mantle cell lymphoma by reducing MCL1.^25^ Meanwhile, the mechanism of Dinaciclib in other diseases has also been studied. CDK1 inhibition by dinaciclib overcomes apoptotic resistance in BRAF‐Mutant Human Colorectal Cancer.^26^ Combined with our research and other studies, it has been shown that Dinaciclib can exert therapeutic effects through different mechanisms in different diseases. However, further efforts were essential to identify more feasible biomarkers in the efficacy of Dinaciclib treatment; combination therapy strategies of Dinaciclib with other CDKs, or chemotherapy regimens should be further evaluated.

In conclusion, we clarified the molecular mechanisms by which dinaciclib induces cell apoptosis and blocks the cell cycle through a *CDK1*‐involved pathway in Raji cells, which supported the view that dinaciclib has a potential value in the treatment of BL.

## CONFLICT OF INTEREST

The authors have declared that no competing interests exist.

## ETHICS APPROVAL

This research included animals and was approved by The First Affiliated Hospital of Zhengzhou University. The study was conducted in accordance with the Guide for the Care and Use of Laboratory Animals.

## Data Availability

The data that support the findings of this study are available from the corresponding author upon reasonable request.
