# Supplementary figures and images for: Study of the mechanism by which dinaciclib induces apoptosis and cell cycle arrest of lymphoma Raji cells through a CDK1‐involved pathway
Source: Cancer Med. 2019 Jun 17;8(9):4348–58. doi: 10.1002/cam4.2324 (PMC6675732; doi:10.1002/cam4.2324)

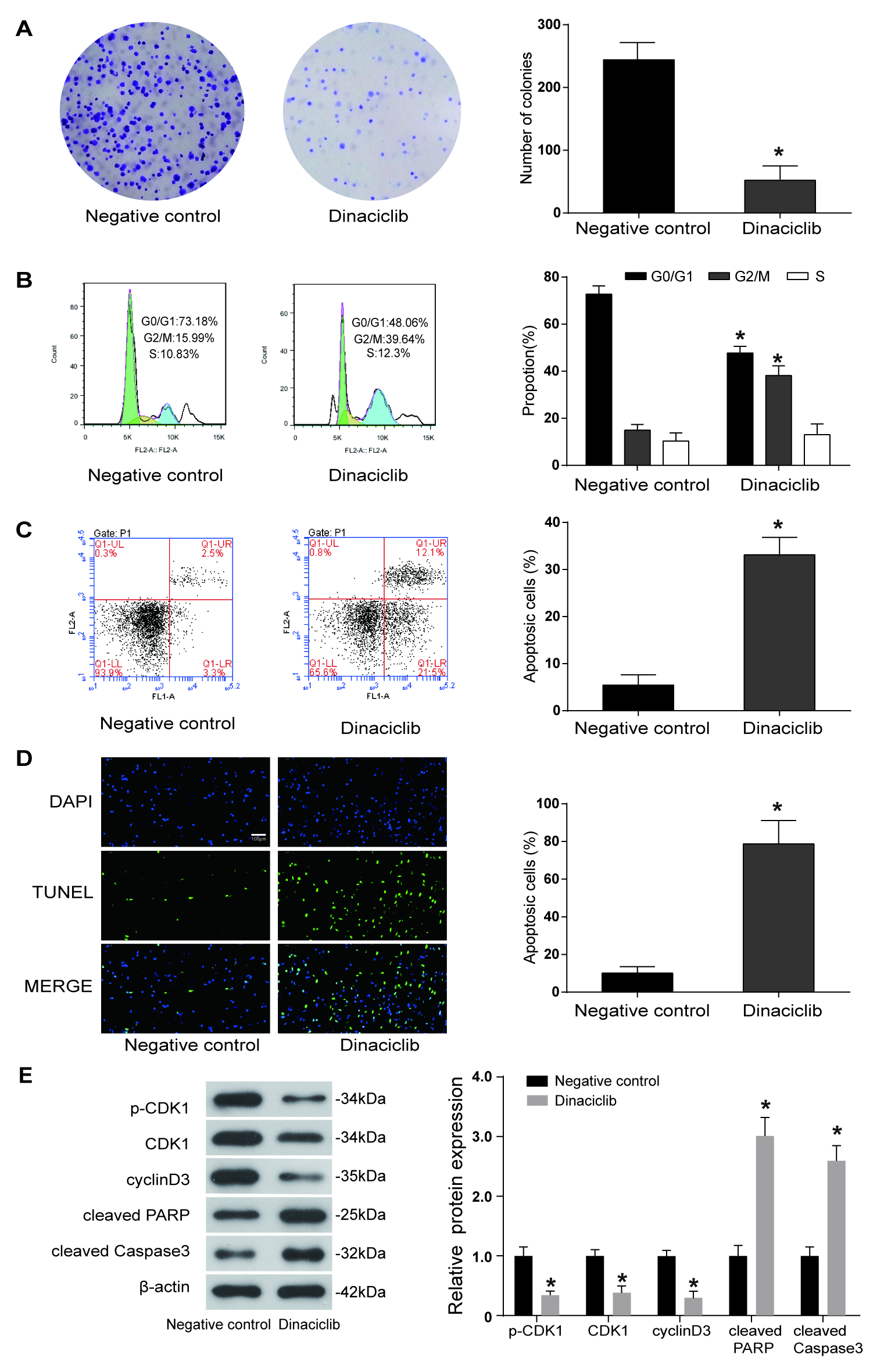

Supplement: Supplementary file 1 [file CAM4-8-4348-s001.tif]
